# A Review of Natural Fiber-Reinforced Composites for Lower-Limb Prosthetic Designs

**DOI:** 10.3390/polym16091293

**Published:** 2024-05-05

**Authors:** Angel D. Castro-Franco, Miriam Siqueiros-Hernández, Virginia García-Angel, Ismael Mendoza-Muñoz, Lidia E. Vargas-Osuna, Hernán D. Magaña-Almaguer

**Affiliations:** 1 Facultad de Ingeniería, Universidad Autónoma de Baja California, Mexicali 21280, Baja California, Mexico; dcastro88@uabc.edu.mx (A.D.C.-F.); virginia.garcia@uabc.edu.mx (V.G.-A.); ismael.mendoza@uabc.edu.mx (I.M.-M.); lidia.vargas@uabc.edu.mx (L.E.V.-O.); 2 Tecnológico Nacional de México, IT de Mexicali, Mexicali 21376, Baja California, Mexico; maganahernan@itmexicali.edu.mx

**Keywords:** natural fiber-reinforced composites, lower-limb prosthesis, mechanical properties, finite-element analysis, biomechanics, polymer matrix composites, ISO standards, fatigue strength, impact resistance, computational modeling

## Abstract

This paper presents a comprehensive review of natural fiber-reinforced composites (NFRCs) for lower-limb prosthetic designs. It covers the characteristics, types, and properties of natural fiber-reinforced composites as well as their advantages and drawbacks in prosthetic designs. This review also discusses successful prosthetic designs that incorporate NFRCs and the factors that make them effective. Additionally, this study explores the use of computational biomechanical models to evaluate the effectiveness of prosthetic devices and the key factors that are considered. Overall, this document provides a valuable resource for anyone interested in using NFRCs for lower-limb prosthetic designs.

## 1. Introduction

Lower-limb amputation is a complex and devastating issue affecting millions of people worldwide [1,2,3,4,5,6]. Prosthetic devices are the most practical choice for restoring ambulatory motor function in individuals with lower-limb amputations [7,8,9]. However, in developing countries, most prosthetic users are compelled to use rudimentary and inefficient designs because of their low acquisition costs [10,11]. This lack of affordable and efficient prosthetic devices perpetuates the marginalization of individuals with lower-limb amputations, subsequently affecting their musculoskeletal system and mobility [12,13,14,15,16,17,18,19,20,21].

In developed countries, the sustainability of materials and manufacturing components used in prosthetic devices is usually not a primary concern in the prosthetic design process [22]. Consequently, the most functional prostheses are those with the most advanced components and naturally higher costs [7,8,10,11,23,24,25,26,27,28]. Therefore, proposing alternative, sustainable, and cost-effective materials for lower-limb prosthetic designs could provide affordable prosthetic solutions for the most vulnerable populations.

Recent advances in natural fiber-reinforced composites (NFRCs) have demonstrated good mechanical and ecological properties compared with synthetic fibers (such as carbon and glass fibers), while also being economically accessible and abundant [29,30,31,32,33,34]. To mention a few, fibers such as kenaf [8,35,36,37], jute [38], ramie [39], and hemp [40] have highlighted their mechanical performance and versatility, comparing favorably against synthetic fibers in terms of their mechanical properties and common applications.

The aim of this review is to methodically evaluate NFRCs (natural fiber-reinforced composites) as a potential substitute material for use in lower-limb prosthetics. This study establishes the groundwork for future research and development in this sector by offering a thorough examination of the characteristics, manufacturing methods, advantages, and drawbacks of NFRCs, along with evaluating the pertinent international standards and available computational biomechanical modeling techniques to validate the performance and usability of devices. This work supports the development and production of prosthetic devices that are affordable, eco-friendly, and high-performing, improving the lives of those who have lost limbs, especially in situations where resources are scarce.

## 2. Background

### 2.1. Lower-Limb Amputation and Prosthetic Options

Lower-limb amputations can be caused by trauma, illnesses, or congenital problems, having a serious negative impact on both physical and emotional health [41,42,43,44,45,46,47,48]. Prosthetic devices have been created to solve this problem, and can be customized to match the unique demands of each user [7,8,9,10,11]. Finding a suitable fit for the user and assuring the durability of the device remain challenges, despite advances in prosthetic technology [18,19,20,21].

One major challenge associated with prosthetic devices is their high acquisition costs, ranging from several thousands to tens of thousands of dollars, depending on the type and complexity of the device. This cost can limit access to prosthetic devices for many individuals, particularly those with inadequate health insurance coverage [10,11,49,50,51,52]. Programs such as Medicare (https://www.medicare.gov, accessed on 12 May 2023) and Medicaid (https://medicaid.gov, accessed on 12 May 2023) in the United States may offer some relief; however, the costs associated with prosthetic devices may not be fully covered. In addition, restrictions on the types of devices covered or the frequency with which they can be replaced may limit access. Nevertheless, it must be highlighted that these government medical programs may vary in their availability and quality from one country to another, or, in some cases, may not exist, especially in developing countries [53].

In low- and middle-income countries, the high acquisition costs of prosthetic devices present a significant barrier to their accessibility [10,49,53,54]. Although efforts have been made to develop low-cost prosthetic devices using alternative materials and manufacturing processes, these devices may not offer the same level of functionality or durability as more expensive options [8,55,56].

### 2.2. Transtibial Prosthetic Design Considerations

Transtibial prostheses are lower-limb prosthetic devices that replace the missing or amputated foot and ankle. The design of transtibial prostheses involves several key components, including the socket, pylon or shank, and the foot or ankle [7,8,27,28,57].

**Figure 1:**
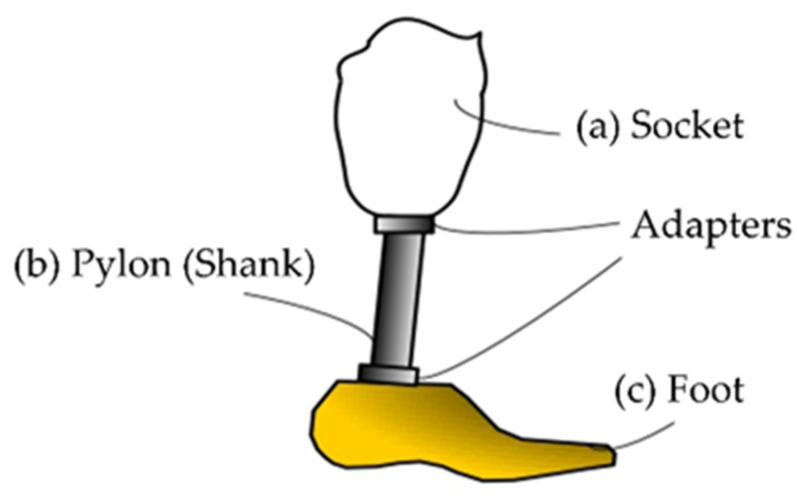
Main components of a passive transtibial prosthesis: (a) the socket, the part of the prosthesis that fits over the residual limb (the remaining part of the amputated leg); (b) the shank (or pylon), a rigid component that extends from the socket down to the foot; (c) the foot, the component of the prosthesis that mimics the function of a natural foot, absorbs shock during walking, and provides stability. Reprinted from ref. [58].

The socket component (Figure 1a) is crucial for the prosthesis to fit the residual limb securely and comfortably. The socket must be made to offer a stable attachment point for the rest of the prosthesis and fit snugly over the residual limb [8,11,27,28]. A good fit is essential to ensure that the prosthesis performs as intended and does not irritate or disturb the user.

Connecting the socket to the foot or ankle component is performed using the pylon or shank component (Figure 1b) of the prosthesis. For weight-bearing activities, such as walking or running [11], this component must provide sufficient support and stability [7]. The materials utilized for this component must be sufficiently sturdy to withstand continuous loading cycles without failure [10].

The foot component (Figure 1c) of the prosthesis is designed to replicate the function of the biological foot as much as possible [59,60,61,62,63,64]. This might involve incorporating features such as shock absorption, flexibility, and articulation into the design [7]. The materials used for this element must also be carefully selected to ensure that they are sufficiently durable to withstand repeated use over time [50]. Table 1 describes some details of the commercial prosthetic feet currently available on the market, as well as the different materials used to manufacture them.

Hydraulic systems, advanced sensors, and control systems are just a few more elements that transtibial prostheses may contemplate if necessary, increasing the cost for the user [11]. These features can provide the prosthesis with more control and allow a more fluid gait pattern.

Transtibial prostheses must be carefully designed considering characteristics such as the proper fit, support, functionality, and material selection [11]. In recent years, advances in both materials science and engineering have achieved more sophisticated prosthetic devices, but to advance the field of prosthetics and improve outcomes for those with amputated lower limbs, continued work in this area is crucial.

### 2.3. Materials Used in Prosthetic Manufacturing

For many years, conventional materials, such as metals and plastics, have mainly been used in the manufacture of prosthetics [7,11,65]. Although they are strong and long-lasting, metals, such as aluminum and titanium, can also be heavy and unwieldy [10,26,66]. In contrast, while plastics such as polypropylene and polyethylene are lightweight, they cannot be as strong or long-lasting as metals and usually deteriorate with time [7,10,65,67,68].

In contrast to conventional materials, synthetic composites offer several benefits. They are created by fusing a matrix substance, such as epoxy resin, with synthetic fibers such as carbon or glass fibers [29,69,70,71,72,73]. Excellent strength-to-weight ratios and customizability for particular design requirements are notable features of synthetic composites [70,74,75,76]. They also exhibit good fatigue characteristics and are corrosion-resistant [77,78,79,80]. Although they can be expensive [71,81,82,83,84,85,86,87], synthetic composites may not be biodegradable [8,27,28].

NFRCs have many advantages over synthetic and conventional materials [30,31,32]. Natural fibers, such as kenaf, flax, or jute, can be used to create these types of composites [29,73,88,89,90]. Natural fiber-reinforced composites have exceptional mechanical qualities, including a low density, biodegradability, and high strength-to-weight and impact–resistance ratios [29,33,34,90]. Additionally, compared with synthetic composites, they are less expensive to manufacture [33].

Ensuring the consistent quality and performance of NFRCs is one of the key issues regarding these materials. Natural fibers can have different qualities depending on factors such as their moisture content and the harvesting conditions [89,91,92,93]. Nevertheless, recent studies have shown that high-quality natural fiber-reinforced composites may be fabricated to match the specifications for prosthetic designs with fiber treatment and a good composite composition [30,31,79,94,95].

Each material has its own unique advantages and disadvantages in prosthetic manufacturing. Conventional materials are durable but may be heavy or uncomfortable for users, and they offer excellent strength-to-weight ratios but may not be biodegradable and can be expensive, whereas natural fiber-reinforced composites offer a lightweight and biodegradable option with excellent mechanical properties but require careful quality control during production.

## 3. Natural Fiber-Reinforced Composites

### 3.1. Characteristics of Natural Fibers

**Figure 2:**
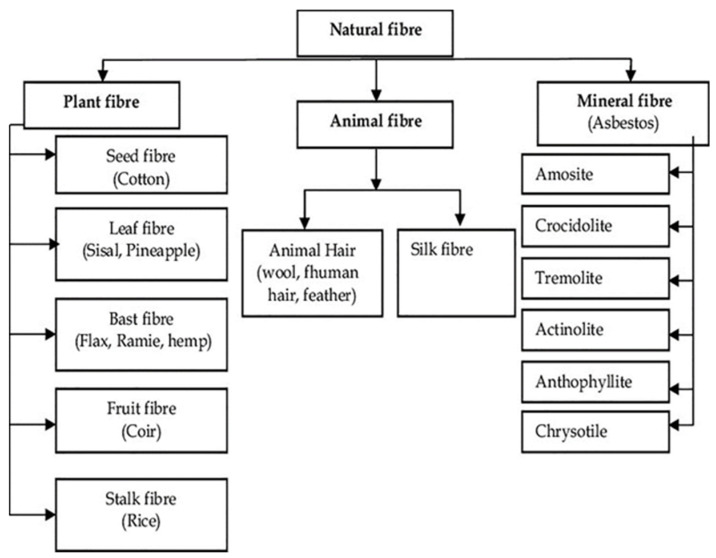
Classification of natural fibers based on their origin: plant, animal, or mineral [29]. Reprinted from Materials Today: Proceedings, Vol. 50, Sandeep Kumar, Alakesh Manna, Rakesh Dang, A review on applications of natural fiber-reinforced composites (NFRCs), Pages 1632–1636, Copyright (2022), with permission from Elsevier.

The utilization of natural fibers as reinforcement materials in composite materials has garnered significant attention owing to their distinctive properties [73]. Derived from diverse sources (Figure 2), such as plants, animals, and minerals [69,90,96], natural fibers have become increasingly popular in recent years owing to their cost-effectiveness, biodegradability, and renewability [30,31,32].

The remarkable characteristic of natural fibers lies in their exceptional strength-to-weight ratio, particularly in jute, flax, and hemp fibers [38,79,90,97]. Owing to their robust tensile strength, natural fibers are highly suitable for integration into composite materials, particularly in applications where weight reduction is paramount, such as the development of lower-limb prosthetics. Furthermore, the low density of natural fibers presents an additional advantage, as they significantly contribute to reducing the overall weight in weight-sensitive applications [29,33,34]. The mechanical and physical properties of the most popular natural fibers are shown in Table 2.

Another noteworthy attribute of natural fibers is their biodegradability and renewability, rendering them an environmentally friendly alternative to synthetic fibers. By incorporating natural fibers into composite materials, the environmental impact of such materials can be reduced, thereby promoting sustainability [8,30,31,32].

Moisture absorption is a significant consideration in the utilization of natural fibers. Untreated natural fibers tend to exhibit higher moisture absorption than treated fibers [95]. This moisture absorption can cause dimensional changes and affect the mechanical properties of composites [33]. Techniques such as alkali treatment and the incorporation of hydrophobic additives or coatings can effectively mitigate moisture absorption [30,31,95].

Natural fibers possess distinctive characteristics that make them highly suitable for integration into composite materials, making them attractive substitutes for synthetic fibers. Incorporating natural fibers into composite materials not only offers the potential to reduce the environmental impact associated with these materials but also provides a sustainable solution for the design of lower-limb prosthetics.

### 3.2. Types and Properties of Natural Fiber-Reinforced Composites

Natural fiber-reinforced composites typically consist of natural fibers embedded within a polymer matrix [96]. The selection of the polymer matrix significantly influences the mechanical properties and overall performance of the composite [29,101,102,103,104,105,106]. The matrix materials commonly used in NFRCs include polypropylene (PP) [40], polyester (PET) [72,107,108], and epoxy [109,110,111,112,113], each offering distinct advantages in terms of their mechanical strength, durability, and compatibility with natural fibers [72,114,115].

While thermoplastic and thermosetting matrices are mostly used in NFRCs, bio-based epoxy resins derived from renewable sources such as vegetable oils offer a sustainable alternative for the matrix phase [116,117,118,119]. These bio-resins can potentially improve characteristics like the biodegradability and environmental impact of NFRCs when combined with natural-fiber reinforcements [118]. However, challenges related to the fiber–matrix compatibility, moisture sensitivity, and achieving the optimal mechanical performance still present difficulties that hinder their use in heavy-duty and long-lasting applications, such as in prosthetic devices.

The mechanical properties of NFRCs play a critical role in determining their suitability for lower-limb prosthetic designs. Parameters such as their tensile strength, flexural strength, impact resistance, and fatigue behavior are of utmost importance and must be carefully considered (Table 1).

Tensile strength represents the maximum stress that a material can withstand under tension before failure. NFRCs exhibit promising tensile strength owing to the reinforcing effect of their natural fibers. Several factors, including the fiber type, fiber content, fiber orientation, and fiber–matrix adhesion, influence the tensile properties of NFRCs [33,69,93,120,121]. Table 3 exhibits a summary of several NFRCs proposed and studied by different authors in detail.

Flexural strength characterizes a material’s ability to resist deformation when subjected to bending. NFRCs exhibit considerable flexural strength, which renders them suitable for load-bearing applications. The flexural properties of NFRCs are influenced by factors such as the fiber content, fiber length, fiber orientation, and matrix properties [33,74,93,141,142]. Attaining proper fiber–matrix interactions and optimal fiber dispersion within the matrix are crucial for achieving enhanced flexural strength.

The impact resistance of NFRCs is of utmost importance in prosthetic design because it determines the material’s capability to absorb and dissipate energy during dynamic loading [7,24,25]. As well as the flexural and tensile strength, the impact properties of NFRCs are influenced by the fiber selection, chemical treatment, and matrix properties [30,31,93]. Comprehensive assessments and optimization of impact resistance are essential to ensure the reliability and functionality of lower-limb prosthetics.

### 3.3. Manufacturing Methods for Natural Fiber-Reinforced Composites

The manufacturing process for natural fiber-reinforced composites (NFRCs) is similar to that of conventional composites, differing primarily in the utilization of natural fibers instead of synthetic fibers. Various manufacturing methods exist for producing NFRCs, each with its own set of advantages and disadvantages [29,33,88,121]. The selection of a specific method depends on its intended application and production requirements [120].

**Figure 3:**
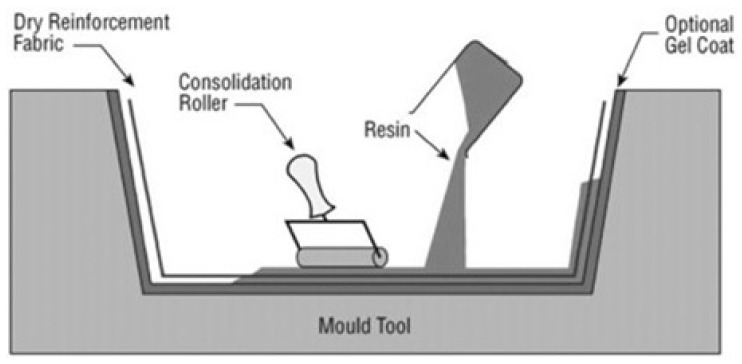
Hand-layup method for manufacturing composite materials [145]. Reprinted from Comprehensive Composite Materials, Author(s): D. Cripps, T.J. Searle, J. Summerscales, Title of chapter: Comprehensive Composite Materials, Pages 737–761, Copyright (2000), with permission from Elsevier.

The hand-layup approach (Figure 3) is the most widely used manufacturing method. It entails manually inserting fibers into a mold, followed by applying resin to help the fibers join. This approach is well suited for small-scale production because it is simple and economical [121,143,144].

Compression molding is another process used in the production of NFRCs. Using this technique, the fibers are placed in a mold and compressed under pressure to release trapped air. The mold is then filled with resin, which is then heated to start the resin’s curing process. Compression molding permits the creation of intricately formed components, and is appropriate for high-volume production [33,89,121,146].

**Figure 4:**
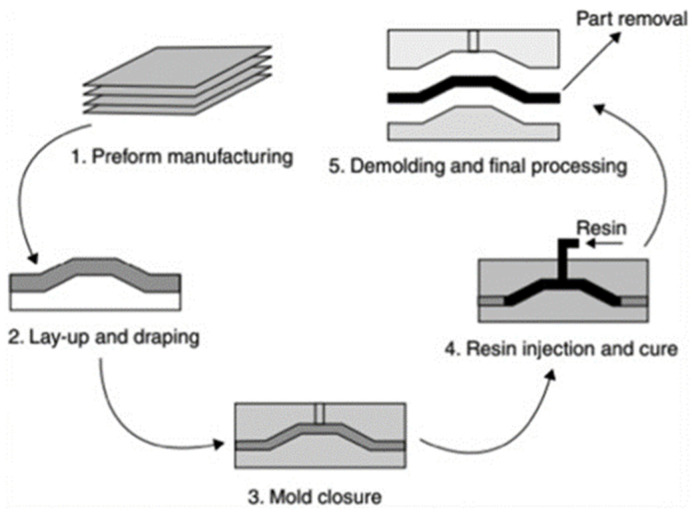
Resin transfer molding (RTM) method for manufacturing composite materials [151]. Reprinted from Design for Sustainability, Author(s): Nadlene Razali, Muhd Ridzuan Mansor, Ghazali Omar, Syed Ahmad Faiz Syed Kamarulzaman, Mohd Hanafee Zin, Nadia Razali, Title of chapter: Design for Sustainability, Pages 395–413, Copyright (2021), with permission from Elsevier.

The resin transfer molding (RTM) method (Figure 4) is employed to produce composites with high strength and stiffness. In this approach, the fibers are positioned within a mold and resin is injected under pressure. The mold is then heated to promote the curing of the resin. RTM has widespread applications in the automotive and aerospace industries [71,147,148,149,150].

Pultrusion is a continuous manufacturing method that is extensively used for the production of fiber-reinforced composites. In this technique, the fibers are pulled through a resin bath and subsequently passed through a heated die, resulting in resin curing. Pultrusion is well suited for creating components with a consistent cross-section and is commonly employed in the construction sector [89,120,121,146,147].

Finally, the filament winding method is employed to manufacture cylindrical NFRC parts, including pipes and tanks. This process involves winding fibers around a mandrel in a specific pattern, followed by the application of a resin to facilitate fiber bonding. After curing, the mandrel is removed, leaving the final product [71,120,146,147,152].

## 4. Lower-Limb Prosthetic Design Using Natural Fiber-Reinforced Composites

### 4.1. Advantages and Drawbacks of Natural Composites in Prosthetic Design

NFRCs have garnered attention in the field of lower-limb prosthetic design owing to their unique properties and potential benefits. However, like other existing materials, they have their own advantages and drawbacks, which must be carefully considered and addressed in the design and manufacturing processes to fully exploit their potential in prosthetic applications.

Advantages:

1. Low Weight and High Strength-to-Weight Ratio: As mentioned before, NFRCs have the remarkable ability to have both a minimal weight and a high strength-to-weight ratio [29,33,34,55]. This quality is very helpful for designing lower-limb prosthetics because it makes it possible to create lightweight, comfortable prosthetics without sacrificing strength and longevity [7,8,9,11,66].
2. Energy Return and Shock Absorption: The mechanical properties of natural composites, including their ability to store and release energy, contribute to enhanced energy return and shock absorption [30,31,55,56]. This feature is crucial in lower-limb prosthetics, as it mimics the natural gait cycle and improves the overall walking efficiency [7,10,24,25,26,58].
3. Sustainability and Environmental Friendliness: Natural fibers used as composite reinforcements, such as kenaf and flax, are renewable resources that provide a sustainable alternative to synthetic fibers [8,29,30,31,32,33,35,36,37,98,119]. The utilization of natural composites in prosthetic design aligns with the increasing demand for eco-friendly materials and reduces the reliance on non-renewable resources.

Drawbacks:

1. Moisture Absorption: Natural fibers have a propensity to absorb moisture [89,93], which can lead to dimensional changes and diminished mechanical properties of the composites [33]. This drawback presents a challenge for prosthetic designs, as exposure to moisture can affect the long-term performance and durability of prosthetic devices [50]. Detailed data on different natural fibers are displayed in Table 1.
2. Limited Durability: Compared to synthetic-fiber-reinforced composites, natural composites may exhibit lower durability and resistance to wear and tear [29,34,153]. The natural fibers used in reinforcement may degrade over time [94], affecting the overall lifespan of prosthetic devices.
3. Variability in Mechanical Properties: Natural fibers, which are organic materials, inherently exhibit variability in their mechanical properties [89,93]. This variability can pose challenges in achieving consistent and predictable performance with natural fiber-reinforced composites in prosthetic designs. This necessitates careful selection and quality control of the natural fibers to ensure consistent mechanical properties and high performance of the prosthetic devices.

### 4.2. Cases of Natural Fiber-Reinforced Composites Used in Prosthetic Designs

**Figure 5:**
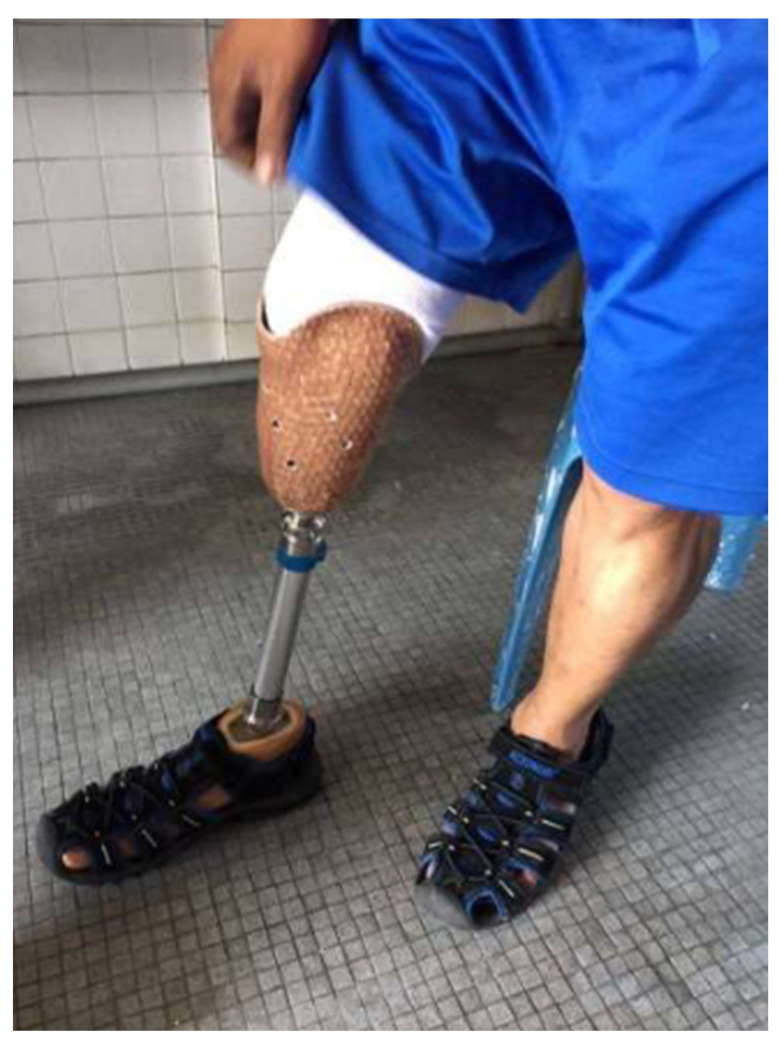
Prosthetic socket made from a kenaf-woven-fabric composite designed by Nurhanisah et al. Reprinted from ref. [8].

Nurhanisah et al. [8] proposed the use of a kenaf-fiber-reinforced composite material for the fabrication of transtibial prosthetic sockets (Figure 5). Their proposed design exhibited favorable results in terms of mechanical properties and comfort, making it suitable for providing good strength and added aesthetic value. Additionally, the proposed design demonstrated environmental friendliness compared to fiberglass-based sockets.

In a study by Irawan et al. [55], the manufacturing of lower-limb prosthetic sockets using ramie fibers and epoxy composites was suggested. Their results showed that such sockets had a significant impact on comfort because of their light weight, strength, and flexibility compared to fiberglass sockets. Furthermore, sockets fabricated with the ramie fibers exhibited a considerably lower weight than those made with fiberglass, with a difference of 186 g, representing a 46.26% reduction. As mentioned several times before, the strength-to-weight ratio exhibited by natural fibers is one of their key advantages over synthetic fibers [33,34], which is essential for maintaining the prosthetic weight within an appropriate range, and thus avoiding increased energy expenditure and excessive stress on the user’s residual limb [11,66].

Moreover, a study by Mankai et al. [56] showed that esparto fibers (*Stipa tenacissima*) are a promising alternative material for manufacturing prosthetic sockets. Their fatigue testing results revealed the viscoelastic behavior of the material and estimated its lifespan to be 2,325,000 cycles, satisfying 77.5% of the ISO 10328 objective.

These studies provide evidence for the potential of NFRCs in lower-limb prosthetic designs. The utilization of these composites can result in lightweight, strong, and biocompatible prostheses. Furthermore, their incorporation into prosthetic designs offers environmental benefits, as natural fibers are renewable resources with a lower carbon footprint than synthetic fibers. This makes them a more sustainable option for prosthetic designs. In addition to their mechanical properties and sustainability advantages, natural fiber-reinforced composites can also provide aesthetic benefits by providing prosthetics with a more natural and organic appearance, thus mitigating the stigma associated with prosthetic use.

## 5. Evaluation of Natural Fiber-Reinforced Composite Prosthetics

### 5.1. Standards and Guidelines for Evaluating Prosthetic Devices

The evaluation of prosthetic devices is an important step in the design process because it ensures that the functional, safety, and performance requirements are met. Adhering to accepted norms and criteria is essential for prostheses constructed of NFRCs to guarantee their safe and efficient use.

One widely recognized standard for evaluating prosthetic devices is ISO 10328, which provides comprehensive guidelines for the mechanical testing of lower-limb prostheses [154,155]. This standard defines the testing procedures for various mechanical properties, including static strength, fatigue strength, and impact resistance. Furthermore, ISO 22675 offers guidelines specifically for testing ankle/foot prostheses, encompassing durability, strength, and range-of-motion requirements [156]. Both ISO 10328 and 22675 establish frameworks for evaluating prosthetic devices, ensuring the adherence to safety and performance requirements. Widely recognized and employed by manufacturers, clinicians, and researchers in the field of prosthetics, these standards guarantee the safety, efficacy, and reliability of prosthetic devices for individuals with lower-limb amputations.

**Figure 6:**
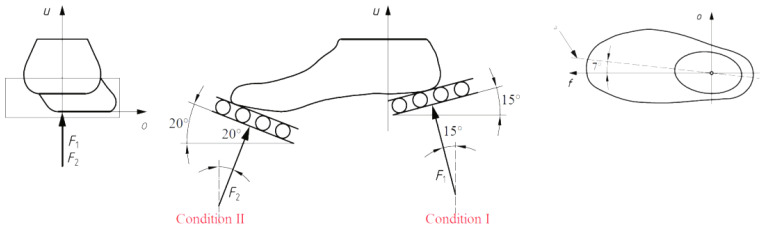
Alignment and angles of test loads for a prosthetic foot, established by ISO 10328 [157]. Reprinted from Procedia Engineering, Vol. 10, C. Colombo, E.G. Marchesin, L. Vergani, E. Boccafogli, G. Verni, Study of an ankle prosthesis for children: adaptation of ISO 10328 and experimental tests, Pages 3510–3517, Copyright (2011), with permission from Elsevier.

ISO 10328 outlines procedures for both the static and dynamic testing of lower-limb prostheses, encompassing mechanical properties such as static strength, fatigue strength, and impact resistance (Figure 6). Static strength testing involves subjecting the prosthetic device to increasing loads until failure, whereas fatigue strength testing simulates cyclic loading to mimic normal usage stresses. Impact resistance testing involves dropping a weight onto the device to simulate the impact of a fall.

**Figure 7:**
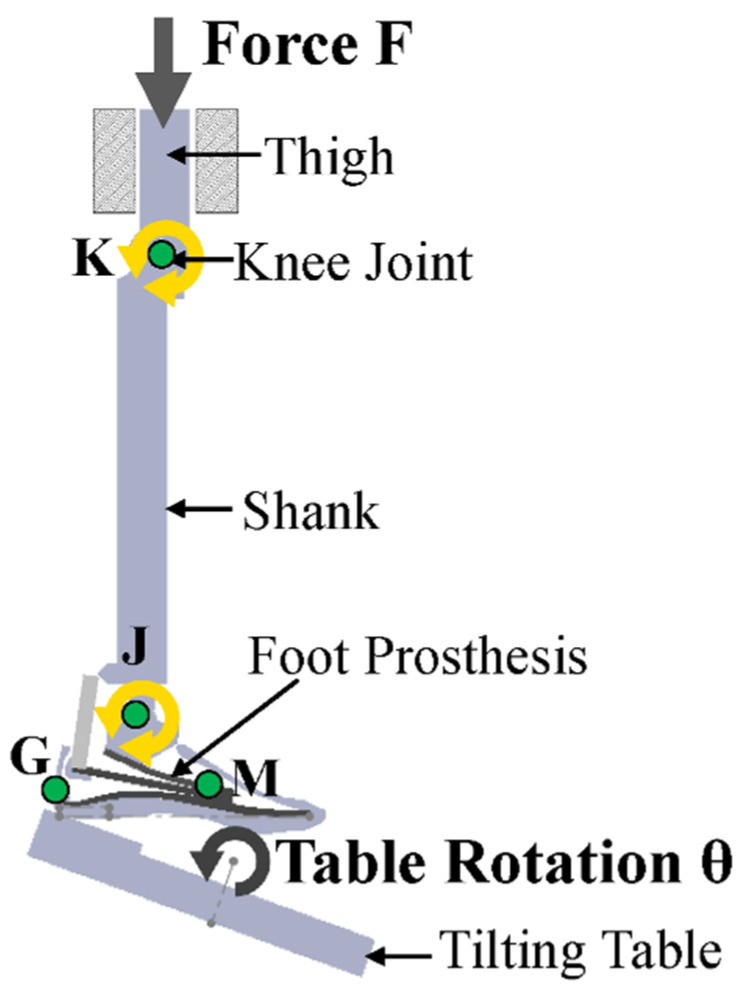
Alignment and angles of test loads for a prosthetic foot established in ISO 22675. Reprinted from ref. [158].

ISO 22675 provides guidelines for testing ankle–foot prostheses, including the requirements for durability, strength, and range of motion (Figure 7). This standard presents testing procedures for both cyclic and static loading, aiming to replicate the load conditions experienced during the stance phase of an individual’s gait. Cyclic loading tests involve applying loads to the device cyclically to simulate typical usage stresses, whereas static loading tests apply loads at specific points in the gait cycle to simulate the maximum loads at those instances.

Numerous other studies have also employed the ISO 10328 [10,56,154,159,160] and 22675 [160,161,162] standards to assess the mechanical properties of prosthetic devices. These standards offer a valuable framework for evaluating the safety and performance of such devices, enjoying broad recognition and adoption by researchers, clinicians, and manufacturers in the prosthetics field. Additionally, organizations such as the American Orthotic and Prosthetic Association (AOPA) (https://www.aopanet.org/, accessed on 1 August 2023) [160,162] and KS P 8403 [163,164] have established guidelines for prosthetic device design, fabrication, fitting, clinical evaluation, and follow-up care.

Although these criteria and recommendations offer a framework for assessing prosthetic devices, it is crucial to keep in mind that not all device types or materials necessarily fall under their purview. Additional testing and assessments may be required for NFRC prostheses to guarantee their compliance with appropriate safety and performance standards.

### 5.2. Using Computational Biomechanical Models to Assess Prosthetic Devices

**Figure 8:**
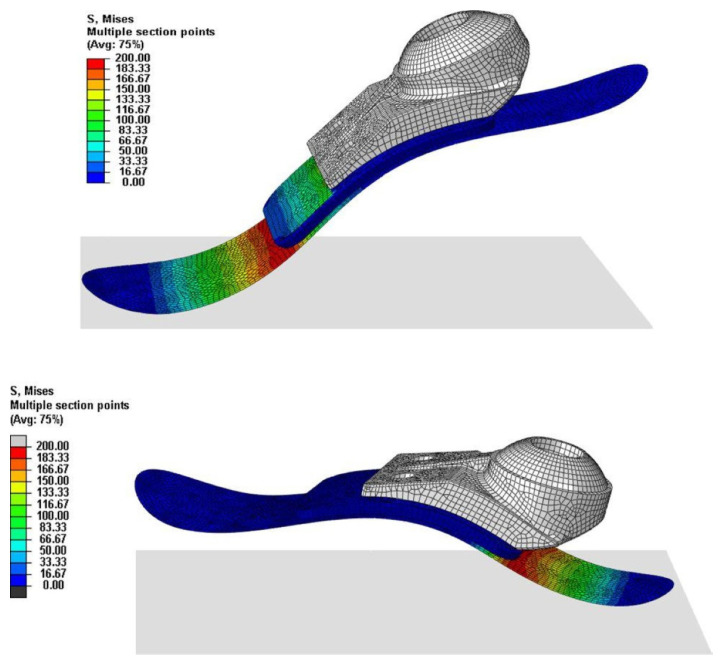
Illustrative finite-element analysis of a prosthetic foot designed and studied by Song et al. [164] (Note: The data shown in this figure is for illustrative purposes only and is not relevant to the present discussion). Reproduced with permission from Youngnam Song et al., International Journal of Precision Engineering and Manufacturing; published by Springer Nature, 2019.

Computational biomechanical models have become a prevalent tool for assessing the performance of prosthetic devices, enabling simulations of the interaction between the device and the residual limb, as well as the forces and stresses during walking. These models offer the opportunity to optimize prosthetic designs and evaluate device performance under diverse conditions [165,166,167] (Figure 8).

**Figure 9:**
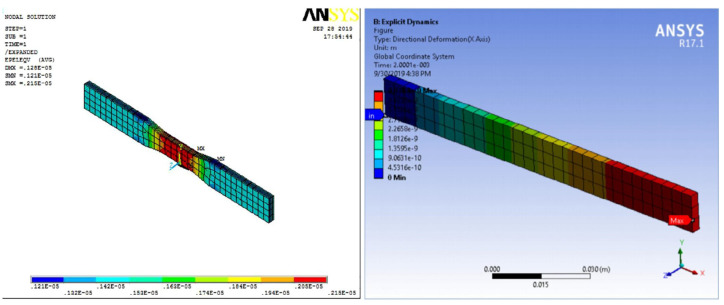
Illustrative tensile and flexural finite-element analysis results for an NFRC studied by Balasubramanian et al. [170] (Note: The data shown in this figure is for illustrative purposes only and is not relevant to the present discussion). Reprinted from Materials Today: Proceedings, Vol. 28, K. Balasubramanian, N. Rajeswari, K. Vaidheeswaran, Analysis of mechanical properties of natural fiber composites by experimental with FEA, Pages 1149–1153, Copyright (2020), with permission from Elsevier.

In the context of NFRC prosthetics, computational biomechanical models allow the assessment of the impact of various design parameters on device performance (Figure 9). Parameters such as the fiber orientation, number of layers, and composite laminate thickness can be evaluated using these models [168,169]. Additionally, these models facilitate assessments of the effects of different loading conditions on device performance, such as walking on different terrains or at varying speeds [165,166,167].

An inherent challenge in using computational biomechanical models for prosthetic designs is the requirement of accurate input data. This includes information about the geometry and material properties of both the prosthetic device and the residual limb. However, advances in imaging technology and material characterization techniques have led to increasingly accurate data acquisition [166,171,172].

The application of computational biomechanical models is a potent tool for designing and evaluating NFRC prosthetics. By utilizing these models, the design of a device can be optimized, and its performance can be evaluated under diverse conditions, ultimately leading to improved prosthetic devices for individuals with lower-limb amputations [67,167,173].

Finite-element analysis (FEA) is a widely used computational biomechanical tool for the design and evaluation of prosthetic devices. FEA is a numerical method that can simulate complex mechanical systems, including prosthetic devices and the human body, by dividing them into simpler elements. The mathematical equations applied to these elements allow FEA to predict the system behavior under different loading conditions [166,174,175]. FEA enables the assessment of mechanical performance in the context of prosthetic designs and evaluation under different specific loading scenarios, including walking, running, and leaping. Additionally, this computational biomechanical tool makes it easier to optimize designs of devices by analyzing the effects of many design factors, such as the material qualities, shape, and thickness.

An advantage of FEA is its ability to assess the stress and strain distribution within the prosthetic device and residual limb. This information helps in identifying areas of high stress or strain that could lead to failure or discomfort, prompting design optimization to mitigate these concerns. FEA has been extensively used in the design and evaluation of NFRCs [120,143,144,162,168,169,170]. For instance, it has been employed to evaluate the impact of different fiber orientations and laminate configurations on the mechanical performance of devices.

Even though computational biomechanics possesses multiple advantages, such as evaluating prosthetic device performance under different loading conditions, there are limitations. The accuracy of the results depends on the accuracy of the input data and assumptions made in the model, which can introduce errors and uncertainties. Acquiring accurate input data, such as the geometry and material properties, may be experimentally challenging. Moreover, the complexity of the models used in computational biomechanics can make interpreting and understanding the underlying mechanisms difficult. Computational biomechanics can also be computationally expensive and time-consuming, which may restrict their use in certain applications. Finally, it is essential to recognize that computational biomechanics cannot fully replace experimental testing, and that the results of computational models should be validated experimentally.

## 6. Future Directions and Conclusions

The integration of NFRCs in lower-limb prosthetic designs has demonstrated significant promise in recent times. These materials have several advantages over conventional prosthetic materials, including enhanced strength, durability, and biocompatibility. Nevertheless, there remains considerable scope for further exploration to fully unlock the potential of these materials.

A compelling avenue for future research is the development of novel NFRC materials with improved properties. This may entail exploring new natural fibers, such as bamboo or hemp, or devising innovative processing techniques to enhance the mechanical properties of existing fibers. Furthermore, it is imperative to evaluate the performance of NFRC prostheses through both computational modeling and experimental testing. This comprehensive assessment is pivotal to ensure the safety and efficacy of these materials across a diverse range of applications.

In conclusion, the integration of NFRCs in lower-limb prosthetic designs holds promise for transformative advancements in the field. These materials exhibit numerous advantages over conventional prosthetic materials, including increased strength, durability, and biocompatibility. Furthermore, they have the potential to offer enhanced cost-effectiveness and eco-friendliness compared to traditional alternatives.

However, to achieve widespread adoption in prosthetic designs, several challenges must be resolved. The foremost task involves the development of new materials with improved properties. Additionally, it is imperative to develop innovative prosthetic devices that effectively capitalize on the distinctive attributes of these materials. Concurrently, ongoing assessments of their safety and efficacy through the combined use of computational models and experimental testing remain crucial.

Notwithstanding these challenges, the potential benefits of NFRCs engender a compelling domain for future research. Through sustained exploration and refinement, these materials could significantly enhance the quality of life of millions of individuals worldwide who rely on prosthetic devices to preserve their mobility and independence.

## Figures and Tables

**Table 1:** Commercial prosthetic feet from different manufacturers.

Brand	Model	Material	Reference (Access Date)	Country
Blatchford	Elan	Carbon Fiber	www.blatchfordmobility.com (17 April 2024)	UK
Blatchford	Epirus	Carbon Fiber	www.blatchfordmobility.com (17 April 2024)	UK
Blatchford	Stellar	Nylon	www.blatchfordmobility.com (17 April 2024)	UK
Össur	Balance Foot S	Glass Fiber	www.ossur.com (17 April 2024)	US
Össur	Vari-Flex	Carbon Fiber	www.ossur.com (17 April 2024)	US
Ottobock	Kintrol	Glass Fiber	www.ottobock.com (17 April 2024)	DE
Ottobock	Restore	Glass Fiber	www.ottobock.com (17 April 2024)	DE

**Table 2:** Physical and mechanical properties of the most popular natural fibers [88,98,99,100].

Fiber	Diameter (μm)	Density (g/cm^3^)	Tensile Strength (MPa)	Young’s Modulus (GPa)	Elongation at Break (%)	Moisture Absorption (%)
Abaca	10–30	1.5	430–813	31.10–33.60	2.9–10	~
Bagasse	~	1.20	20–290	19–27	1.10	~
Bamboo	88–125	0.91–1.26	503	35.91	1.40	~
Banana	100–250	1.35	529–914	27–32	2.60–5.90	~
Basalt	17	2.8	4800	90	3.15	~
Coconut	150–250	1.15–1.25	131–220	4–6	15–40	10
Cotton	~	1.50–1.51	287–597	5.50–12.60	0.30–10	8–25
Flax	25	1.40–1.50	345–1500	27.60–80	1.20–3.20	7
Hemp	25–600	1.48–1.50	550–900	70	1.60–4	8
Henequen	~	1.20–1.40	430–570	10–16.30	3.70–5.90	~
Jute	25–250	1.30–1.48	393–800	0.13–27.60	1.16–1.80	12
Kenaf	~	1.25–1.40	284–930	0.13–26.50	1.16–1.80	~
Pineapple	50	1.44	413–1627	60–80	14.50	~
Ramie	20–280	1.30–1.50	400–938	61.40–128	3.60–3.80	12–17
Rice husk	~	0.50–0.70	~	~	~	~
Sisal	50–200	1.30–1.50	390–635	9.40–41	2–2.50	11
Softwood	~	1.50	1000	40	~	~
Viscose cord	~	~	593	11	11.4	~

**Table 3:** Tensile and flexural strengths of some NFRCs [68].

Authors	Matrix	Types of Fiber	Method of Fabrication
Aslan et al. [122]	Polypropylene matrix	Carbon/sisal, glass/sisal	Single-screw co-rotating extrusion method
Assarar et al. [123]	Epoxy matrix	Flax–carbon fiber	Platen press process
Campbell et al. [124]	Plant oil resin	Ramie/stockinet	Standard layup method
Chaudhary et al. [125]	Epoxy matrix	Flax hemp/jute/fiber	Hand-layup method
Essabir et al. [126]	Polypropylene matrix	Coir fiber	Twin-screw extrusion method
Gu et al. [127]	Epoxy matrix	Ramie fiber	Vacuum infusion process
Indra Reddy et al. [128]	Epoxy matrix	Pineapple, glass, and jute fiber	Hand-layup method
Jagannatha et al. [129]	Epoxy matrix	Glass/carbon	Vacuum bagging technique
Lee et al. [130]	Polypropylene matrix	Kenaf/jute	Hot-pressing method
Rahman et al. [131]	Vinyl-ester matrix	PALF	Hand-layup method
Saba et al. [132]	Epoxy matrix	Kenaf fiber	Hand-layup technique
Sekaran et al. [133]	Epoxy matrix	Sisal fiber and aloe vera	Hand-layup method
Shanmugam et al. [134]	Polyester matrix	Jute fiber palmyra and leaf stalk fiber	Compression molding
Shih et al. [135]	Poly-lactic acid	PALF/chopsticks	Counter-rotating internal mixing
Shrivastava et al. [136]	Epoxy resin	Coir–glass	Hand-layup method
Sreekumar et al. [137]	Polyester matrix	Sisal fiber	Resin transfer molding technique
Widhata et al. [138]	Methyl methacrylate	Water hyacinth	Compression molding
Yan et al. [139]	Epoxy matrix	Flax/linen/bamboo	Vacuum bagging process
Yang et al. [140]	Polypropylene matrix	Hemp fiber	Twin-screw extrusion
